# Circulating Hemocytes from Larvae of the Japanese Rhinoceros Beetle *Allomyrina dichotoma* (Linnaeus) (Coleoptera: Scarabaeidae) and the Cellular Immune Response to Microorganisms

**DOI:** 10.1371/journal.pone.0128519

**Published:** 2015-06-01

**Authors:** Sejung Hwang, Kyeongrin Bang, Jiae Lee, Saeyoull Cho

**Affiliations:** Department of Applied Biology, College of Agriculture and Life Science, Environment Friendly Agriculture Center, Kangwon National University, Chuncheon, Republic of Korea; Institute of Oceanology, Chinese Academy of Sciences, CHINA

## Abstract

Hemocytes of the last larva of the Japanese rhinoceros beetle *A*. *dichotoma* (Linnaeus) (Coleoptera: Scarabaeidae) were classified as granulocytes, plasmatocytes, oenocytoids, spherulocytes, prohemocytes, and adipohemocytes. Among these cell types, only the granulocytes became immunologically activated with obvious morphological changes, displaying large amoeba-like, lobopodia-like, and fan-like structures. In addition, their cytoplasmic granules became larger and greatly increased in number. To explore whether these granules could be immunologically generated as phagosomes, total hemocytes were stained with LysoTracker. Greater than 90% of the granulocytes retained the LysoTracker dye at 4 h post-bacterial infection. In flow cytometry analysis, the red fluorescent signal was highly increased at 4 h post-bacterial infection (60.36%) compared to controls (5.08%), as was confirmed by fluorescent microscopy. After 12 h post-infection, these signals returned to basal levels. The uptake of pathogens by granulocytes rapidly triggered the translocation of the microtubule-associated protein 1 light chain 3 alpha (LC3) to the phagosome, which may result in enhanced pathogen killing.

## Introduction

Although invertebrates do not have an antibody-based immune system (a form of adaptive immune response in vertebrates), invertebrates do have an innate immune system. The insect immune system can be divided broadly into two classes, cellular and humoral immune reactions. The humoral immune response is based on antimicrobial peptides (AMPs), which are synthesized primarily in the fat body and destroy invading pathogens. As described in detail previously, many pathways are involved in the production of AMPs, including Toll/Toll-like receptors (TLRs), the immune deficiency (Imd) pathway, and the Janus kinase (JAK)/signal transducers and activators of transcription (STAT) signaling [1], [2], [3].

However, insect cellular immune reaction is characterized by the immune activation of insect blood cells themselves (hemocytes). Immunologically activated hemocytes directly kill pathogens through well-known cellular defensing processes such as phagocytosis, encapsulation, and nodulation [3]. The cellular immune reaction is initiated when insect pattern recognition receptors (PRRs) recognize invasive microorganisms. Insect PRRs such as C-type lectins, peptidoglycan recognition proteins (PGRPs), Gram-negative binding proteins (GNBPs), and nucleotide binding oligomerization (NOD)-like or retinoic acid inducible gene (RIG)-like receptors recognize microbe-associated molecular recognition patterns (MAMPs) expressed by pathogens, including microbial peptidoglycans, lipopolysaccharide, β-glucans, lipoproteins, CpG dinucleotides, and flagellin. This is followed by activation of downstream signaling and an effector response [4]. Although the downstream signaling molecules and effectors of the insect cellular immune reaction are less well known than that in mammals, insects also have homologs of cellular immune-related proteins such as focal adhesion kinase (Fak), protein tyrosine kinase2 (Pyk2), and paxillan (DpaxA), and they likely play similar roles in the insect cellular immune reaction [5]. Both phosphoinositol-3-kinase (PI3K) and Ras-related C3 botulinum toxin substrate (Rac) signaling activity were observed and associated with the cellular immune reaction in *Drosophila* [5], [6]. The insect zymogen prophenoloxidase (PPO) was also shown to be an important component of the cellular immune reaction by its ability to induce insect hemolymph melanization [7]. The PPO zymogen is cleaved by a serine protease cascade, and then is converted into its activated form, phenoloxidase (PO), which oxidizes phenolic molecules to produce melanin around invading pathogens and wounds [7]. Several studies demonstrated that phagocytosis and nodule formation were substantially reduced by knock-down of PPO in *Aeromonas hydrophila* [8].

As mentioned above or previously [3], insect hemocytes play an important role in cellular immunity, and identification of types of hemocytes is important for understanding the cellular immune system in insects [9]. Insect hemocytes have been characterized and identified by their morphology, protein expression, and functional characteristics [10]. Insect hemocytes are categorized functionally as prohemocytes, plasmatocytes, granulocytes, spherulocytes, adipohemocytes, coagulocytes, and oenocytoids [11], [12]. Among these cell types, plasmatocytes and granulocytes are generally considered as key players in the cellular immune system [3]. These two types of hemocytes undergo dramatic changes in morphology when they encounter pathogens, and have been observed engulfing and killing pathogens by phagocytosis. As described in detail previously [3], our studies revealed that the granulocytes in the larvae of the beetle *Protaetia brevitarsis seulensis* play a pivotal role in cellular immune responses and are specialized to perform specific functions, such as phagocytosis and encapsulation. These cells were also observed to carry out autophagy-related phagocytosis [3]. In mosquitos, granulocytes are also the most abundant hemocyte and play a pivotal role in immune responses [13]. Plasmatocytes are also the most abundant cell type in flies and are specialized to kill pathogens [14]. Although granulocytes are considered the main phagocytes in *P*. *brevitarsis seulensis* beetle larvae, we also observed that plasmatocytes occasionally engulfed bacteria and yeast. Likewise, both granulocytes and plasmatocytes are involved in cellular immunity in most Lepidoptera and some Coleoptera [15], [16]. As described in detail previously [3], this study also reported here characterized hemocytes and the cellular immune reaction in the Japanese rhinoceros beetle *Allomyrina dichotoma* (Linnaeus) (Coleoptera: Scarabaeidae). Although this beetle spends its adult life in trees eating sap and fruits, this represents less than 4 months of its life; most of its lifespan is spent as an egg, larva, or pupa that lives on the damp group and must fight off numerous microorganisms. Therefore, we also speculated that which beetle would have a powerful cellular immune system, which we attempted to characterize in this study. Six types of hemocyte were identified based on their size, morphology, and dye-staining properties [11], [13], [16], [17]. Among these hemocytes, granulocytes were found to be the key facilitators of the cellular immune reaction. In addition, granulocytes were observed to be very specialized to perform specific functions, such as phagocytosis and nodulation, and also carried out autophagy-related phagocytosis.

## Results

### Morphological characteristics of hemocytes

**Fig 1:**
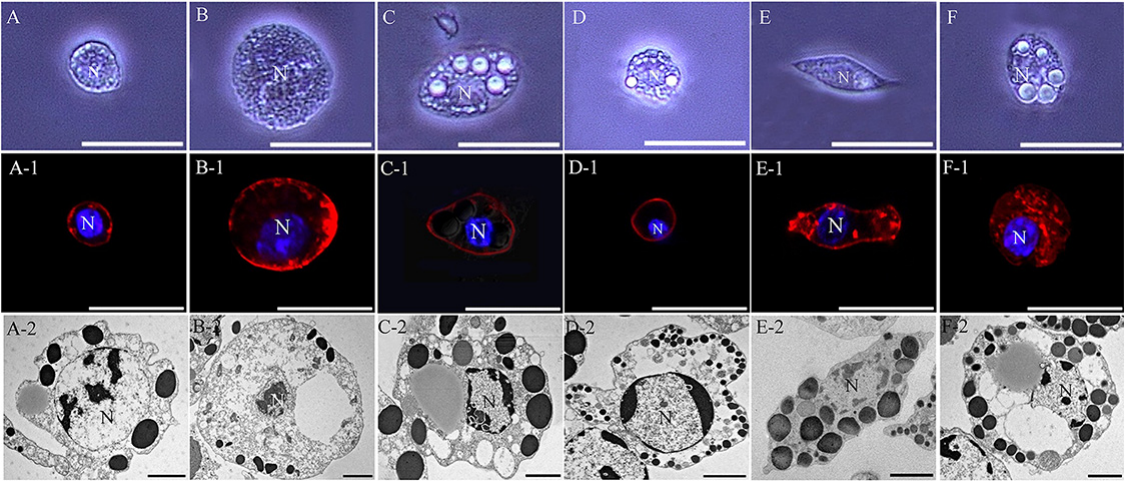
Image of hemocytes. Hemocytes were classified as prohemocytes (A, A-1, and A-2), oenocytoids (B, B-1, and B-2), adipohemocytes (C, C-1, and C-2), spherulocytes (D, D-1, and D-2), plasmatocytes (E, E-1, and E-2), and granulocytes (F, F-1, and F-2) on the basis of their size and morphology. Confocal images of hemocytes stained with DAPI (blue) to label nuclei and antibodies to filamentous actin (F-actin; red) to label the cytoskeleton. N, nucleus. (A-F1) Scale bar = 20 μm, (A2-F2) Scale bar = 2 μm.

**Fig 2:**
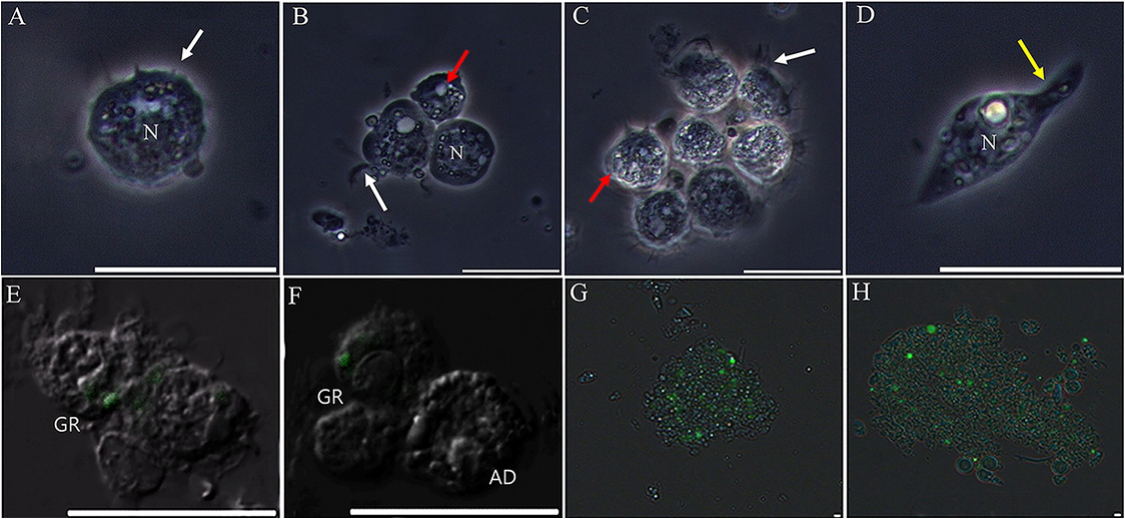
Image of immunologically activated granulocytes, including phagocytosis and nodulation. (A, B, and C) The granulocytes changed their shape and generated lodopodia-like or fan-like structures (indicated by white arrow). The vacuoles expanded in the cytoplasm (indicated by red arrows). Several filopodia were also generated in plasmatocytes (D; indicated by yellow arrows). The granulocytes phagocytosed and engulfed GFP-expressing yeast at 30 min post-infection (E and F). Nodulation by granulocytes at 30 min post-bacterial infection (G and H). N, nucleus; GR, granulocyte; AD, adipohemocyte. Scale bar = 20 μm.

As described in detail previously [3], the hemocytes of the last larva of the Japanese rhinoceros beetle *Allomyrina dichotoma* (Linnaeus) (Coleoptera: Scarabaeidae) were also classified as six types: granulocytes, plasmatocytes, oenocytoids, spherulocytes, prohemocytes, and adipohemocytes. As shown in Fig 1, inverted fluorescence microscopy, confocal microscopy, and transmission electron microscopy (TEM) were used to characterize the hemocytes’ morphology, and the cells were classified based on previously described criteria [3], [18]. The prohemocytes were the smallest round cells in the hemolymph, displaying an average width of 6.17 μm and an average length of 6.49 μm (n = 20), and were characterized by a high nucleus/cytoplasm ratio (Fig 1A, 1A-1 and 1A-2). The cytoplasm was almost filled with the nucleus, and scattered heterochromatin was observed in the nucleus. Several large electron-dense, dark granules were occasionally observed in cytoplasm (Fig 1A-2). The oenocytoids were larger, round hemocytes (average width, 21.66 μm, and average length, 19.30 μm; n = 20) found in the hemolymph, with small nuclei with a well-developed nucleoli (Fig 1B, 1B-1 and 1B-2). These cells had few membrane-bound granules as compared with other hemocyte types; however, large transparent vacuoles, including small, electron-dense dark granules, were frequently observed in their cytoplasm (Fig 1B-2). The adipohemocytes were large, oval cells (average width, 11.45 μm, and average length, 19.34 μm; n = 20) (Fig 1C, 1C-1 and 1C-2). Their nuclei were relatively small, and numerous small and large electron-dense dark granules and transparent/translucent vacuoles, which appeared like large lipid droplets, were observed in the cytoplasm (Fig 1C-2). The spherulocytes were relatively small round cells (average width, 9.02 μm, and average length, 7.72 μm; n = 20) with many small granules in the cytoplasm (Fig 1D, 1D-1 and 1D-2). They usually contained a round, large nucleus with condensed chromatin (Fig 2D-2). The plasmatocytes had a typical spindle shape (average width, 7.28 μm, and average length, 18.38 μm; n = 20) (Fig 1E, 1E-1 and 1E-2) and had a lobated nucleus with finely distributed chromatin. As described in many studies, the plasma membrane of the plasmatocytes exhibited micropapillae, long filopodia, or other irregular structures (Fig 1E-2). The granulocytes were medium-sized (average width, 17.03 μm, and average length, 15.08 μm; n = 20), round or slightly oval cells with many small and several large granules in their cytoplasm (Fig 1F, 1F-1 and 1F-2). At first glance, these cells appeared to be very similar to the adipohemocytes in morphology. However, granulocytes can be distinguished from adipohemocytes because they are smaller and rounder than adipohemocytes (Fig 1C and 1F), and several larger granules were frequently observed in their cytoplasm. These granules seem to be different from those observed in adipohemocytes because they are larger and more translucent (Fig 1F and 1F-2). In addition, their plasma membrane was sometimes irregular and contained occasional micropapillae, while the plasma membrane of the adipohemocytes was smooth, without any membrane structures. Therefore, we classified this cell type as granulocyte and we confirmed this result by pathogen exposure.

### Hemocyte types involved in the cellular immune reaction

To confirm which of the six types of hemocytes were immunologically associated, we conducted *in vivo* assays by injecting the larva with *E*. *coli* and *S*. *cerevisiae*. At 12 h post-bacterial infection, only the granulocytes became immunologically activated, with dramatic changes in morphology. After pathogen exposure, these cells displayed large, amoeba-like, lobopodia-like, and fan-like (indicated by white arrows) structures, and the size and number of cytoplasmic granules were greatly increased (indicated by red arrows) (Fig 2A, 2B and 2C). Some plasmatocytes also changed shape, but there were no sudden or rapid changes in morphology and no changes in the polymorphic granules in their cytoplasm (Fig 2D). The same results were also observed 18 h post-infection (data not shown). No morphological changes were observed in the other cell types (prohemocytes, oenocytoids, adipohemocytes, and spherulocytes) compared to naïve larvae hemocytes (data not shown). To reconfirm that the granulocytes were the key cell type involved in the cellular immune response, green fluorescent protein (GFP)-expressing bacteria and yeast were injected into larvae. Phagocytic activity was verified by visualization of GFP-expressing bacteria and yeast inside the granulocytes (Fig 2E and 2F). In addition, many granulocytes were observed to conjugate with GFP-expressing bacteria and yeast, similar to what occurs during the nodulation process (Fig 2G and 2H). As expected, we did not observe any immunological activity in any other cell types except for a small number of plasmatocytes. Taken together, these results indicated that the granulocytes are the main immunological cell in the last larva of the Japanese rhinoceros beetle.

### Effect of infection on hemocyte number

**Fig 3:**
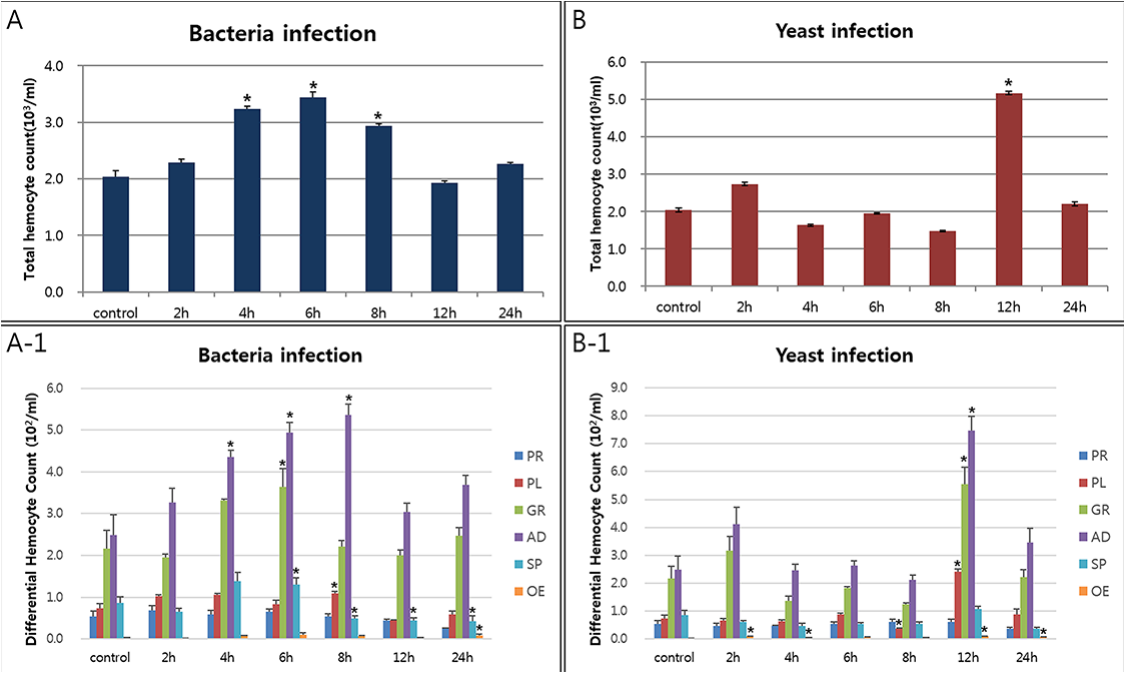
Total hemocyte and average proportions of hemocytes in native and challenged larvae. Total hemocyte counts (A and B) and differential hemocyte counts (A-1 and B-1) were performed. The number of hemocytes of the six circulating hemocyte types were counted in 39 larvae (33,270 hemocytes). Each group (control larvae or 2, 4, 6, 8, 12, or 24 h post-infection) contained three larvae. Results are given as the mean and standard deviation.*(P<0.05) Challenged larvae were infected with bacteria (panel A and A-1) or yeast (panel B and B-1). PR, prohemocytes; PL, plasmatocytes; GR, granulocytes; AD, adipohemocytes; SP, spherulocytes; OE, oenocytoids.

To determine whether there is any relationship between hemocyte number and pathogen infection, circulating hemocyte numbers [total hemocyte count (THC), cells/ml] and relative percentages [differential hemocyte count (DHC)] were measured in the control group and in the bacteria- and yeast-challenged larvae (Fig 3A, 3A-1, 3B and 3B-1). Approximately 30,000 hemocytes from 39 larvae were counted at each time point (2 h, 4 h, 6 h, 8 h, 12 h, and 24 h). The initial number of circulating hemocytes (control group) was 4.1 ± 0.21 × 10^3^ /ml (n = 3), which increased to 6.9 ± 0.20 × 10^3^ /ml (n = 3) after 6 h of bacterial infection (Fig 3A). There were significant differences in THC between 0 h and 4 h, 6 h, and 8 h post-bacterial infection, and THC returned to baseline levels after 8 h post-infection (Fig 3A). The relative concentration number of each type of hemocyte in control and bacterial-challenged larvae are shown in Fig 3A-1. The concentration number of every cell type was slightly increased after bacterial infection. Among the six types of hemocytes, the adipohemocytes, granulocytes, and spherulocytes were significantly different between control and bacterial-challenged larvae (Fig 3A-1). For larvae challenged with yeast, differences in the THC and DHC were similar to those observed in larvae challenged with bacteria (Fig 3B and 3B-1).

As described in detail previously [3], although the concentration number of adipohemocytes or spherulocytes was significantly different between control and challenged larvae at specific times (P < 0.05; χ^2^), we did not observe any evidence that the adipohemocytes might have differentiated into other cell types. In addition, we did not observe any substantial or rapid increase in the number of granulocytes during pathological progression.

### LysoTracker analysis of granulocytes

**Fig 4:**
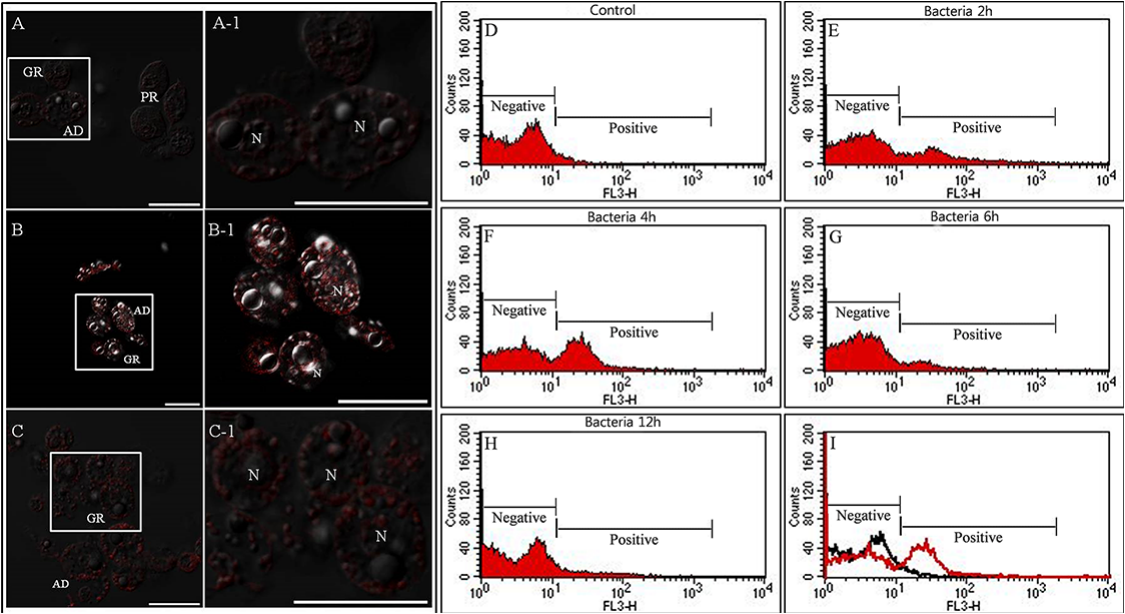
LysoTracker Red labeling of lysosomes in granulocytes and flow cytometric analysis after bacterial infection. (A and A-1) 0 h post injection, (B and B-1) 4 h post injection, (C and C-1) 12 h post injection. A1, B1, and C1 indicate a higher magnification of the regions in inset of panel A, B, and C. Greater than 90% of the granulocytes were stained with LysoTracker at 4 h post-bacterial infection, while very little LysoTracker staining was observed in control larvae. The red signal returned to baseline at 12 h post-infection. (D through H) flow cytometric analysis at 0 h, 2 h, 4 h, 6 h, and 12 h post injection. Based on the red fluorescence intensity, two peaks were identified, negative and positive peak. (I) The black line histogram indicates control larva hemocytes and the red line histogram indicates bacterial-challenged larva hemocytes at 4 h post injection. N, nucleus; GR, granulocytes; AD, adipohemocytes. Scale bar = 20 μm.

As described above, the granulocytes and the adipohemocytes were characterized by highly polymorphic, large granules in their cytoplasm. However, the granules in each cell type were morphologically different (Fig 1C and 1F). To explore whether these granules could be immunologically generated as phagosomes, total hemocytes were stained with LysoTracker 2 h, 4 h, 6 h, and 12 h post-bacterial infection. Greater than 90% of the granulocytes and a small number of plasmatocytes were stained with LysoTracker at 4 h post-bacterial infection, while very little LysoTracker staining was observed in control larvae (Fig 4A, 4A-1, 4B and 4B-1). The other cell types, including adipohemocytes, did not stain with LysoTracker during the process of infection. At 4 h post-infection, the peak staining intensity was observed in the granulocytes, and the signal returned to baseline at 12 h post-infection (Fig 4C and 4C-1). To quantify these signals, the florescence intensity was analyzed by flow cytometry. As shown in Fig 4D through to 4H, the red fluorescent signal was highly increased at 4 h post-bacterial infection (60.36%) compared to control samples (5.08%), as was observed by fluorescent microscopy. After 12 h post-infection, the signal returned to the control state (Fig 4H). To compare the red fluorescent signal intensity between controls and infected samples at 4 h post-infection, a representative histogram overlay is shown (Fig 4I). The black line indicates the fluorescent intensity of control hemocytes and the red line indicates the fluorescent intensity of hemocytes at 4 h post-bacterial infection. These results indicate that the granulocytes were the main immunological cell in these larvae and that their large granules were phagosomal or lysosomal compartments involved in the process of pathogen degradation.

### Autophagy-related phagocytosis by granulocytes

**Fig 5:**
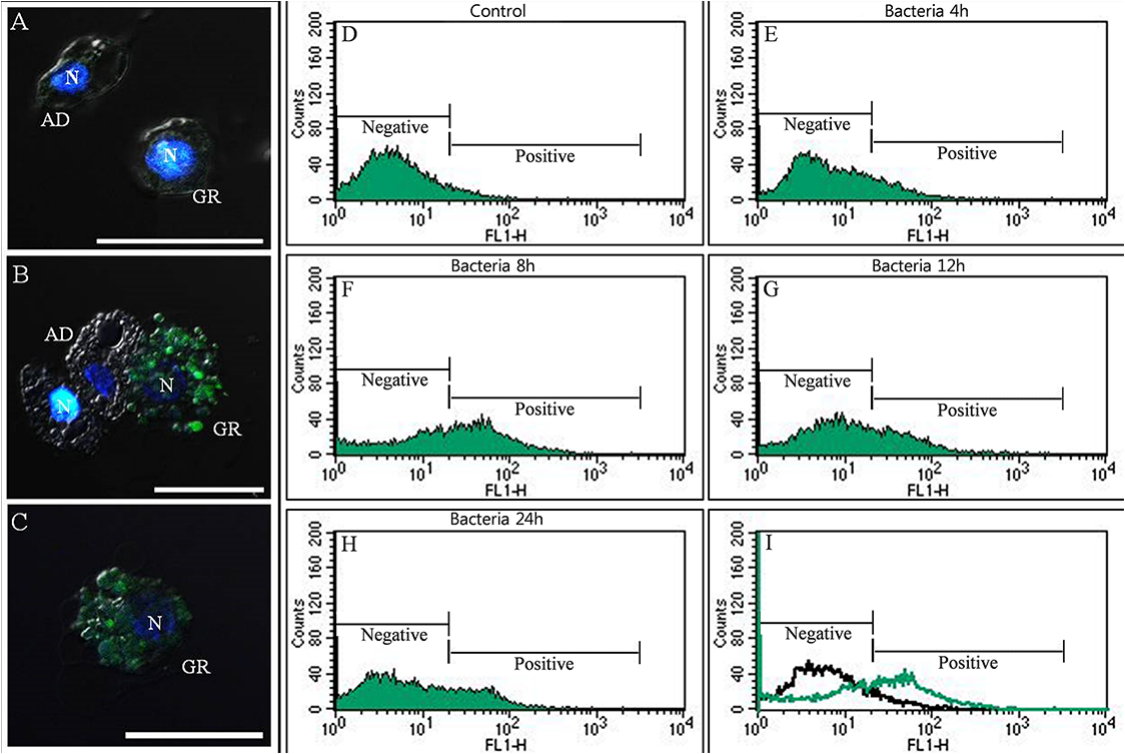
GFP-LC3 labeling in granulocytes and flow cytometric analysis after bacterial infection. A Cyto-ID Autophagy Green dye reagent was used for the detection of autophagosome formation in granulocytes at 0, 4, 8, 12, and 24 h post infection. (A, B and C) Confocal Images of granulocytes stained with DAPI and GFP-LC3 (A; 0 h post injection, B; 8 h post injection, C; 24 h post injection); and flow cytometric analysis at 0, 4, 8, 12, and 24 h (D through H). After 8 h of infection, positive staining was observed in the granulocytes (B) and the GFP signal was weakly maintained at 24 h post infection (C). Based on the green fluorescence intensity, two peaks were identified, negative and positive peak. The increase in GFP-LC3 staining at 8 h post infection (F). (I) Representative histogram overlay (black line; 0 h post infection, green line; 8 h post infection). N, nucleus; GR, granulocytes; AD, adipohemocytes. Scale bar = 20 μm.

As described in detail previously [3], we demonstrated that highly activated hemocytes expressed the microtubule-associated protein 1 light chain 3 alpha (LC3), which is associated with the process of the autophagy [3]. To determine whether pathogenic phagocytosis in granulocytes was also related to autophagy in this beetle, hemocytes were stained with GFP-LC3, a widely used marker of autophagosome formation, and analyzed by fluorescent microscopy and flow cytometry. Every cell type was negative for GFP-LC3 before bacterial infection. After 8 h of infection, positive staining was observed in the granulocytes, but not in the other hemocytes, of bacterially infected larvae, and the GFP signal was maintained at 8 h post-infection (Fig 5A, 5B and 5C). In addition, flow cytometric analysis confirmed the increase in GFP-LC3 staining at 8 h post-infection (Fig 5D–5H). At 8 h post-infection, 47.81% of granulocytes (compared to 5.84% of granulocytes in controls) were positive for GFP (Fig 5A and 5C). These results demonstrated that granulocytes might play a role in autophagy-related pathogenic processes.

## Discussion

As described in detail previously [3], this study also aimed to classify and identify immune cells in the circulating hemocytes of the Japanese rhinoceros beetle *Allomyrina dichotoma*. As shown in Fig 1, six hemocyte types were identified and classified as prohemocytes, oenocytoids, spherulocytes, adipohemocytes, plasmatocytes, and granulocytes, based on their morphology and function. Among these cell types, the granulocytes were revealed to be professional phagocytes in this insect. Phagocytosis was first described by Eli Metchnikoff and is known as an evolutionarily conserved cellular immune response in multicellular organisms [19]. As described in detail previously [3], general phagocytes could be characterized by the presence of pseudopodia or filopodia on their plasma membrane after immunological activation. Thus, to identify professional phagocytes, cell types were examined morphologically for the presence of fan-like structures or networks of extracellular fibers, which become pseudopodia or filopodia after pathogen exposure. As shown in Fig 2, the generation of fan-like structures on the granulocyte plasma membrane was observed, and these structures were dramatically extended in the presence of pathogens and were used for capturing yeast and bacteria *in vivo*. In addition, nodulation was also mainly performed by granulocytes.

At first glance, the granulocytes appeared to be morphologically very similar to the adipohemocytes because both cell types have several large granules in their cytoplasm. As described in detail previously [3], during the process of hemocyte classification, we observed that pseudopodia or filopodia actively formed only on the granulocyte plasma membrane during infection, while the adipohemocyte plasma membrane remained smooth, without the formation of any membrane structures. In addition, plasmatocytes, which are well known as insect phagocytes, did not exhibit a dramatic change in morphology after immune challenge and did not actively phagocytose bacteria or yeast. These results indicate that granulocytes are the main professional phagocyte in this insect. In flies and mosquitos, plasmatocytes and granulocytes are the most abundant cell types in circulating hemocytes and are professional phagocytes [13], [14]. Although there are some exceptions, plasmatocytes and granulocytes are the main phagocytic cells in most insects [13], [15], [19], [20], [21].

The insect cellular immune response is characterized by a variety of immune functions of hemocytes such as phagocytosis, melanization, encapsulation, and coagulation [17]. Researchers must classify and identify the immune cells capable of killing pathogens to establish the evolution of the cellular immune response in multicellular organisms, including insects. Therefore, hemocyte classification has long been a key area of focus in the study of the insect cellular immune response. However, insect hematology, including hemocyte classification, is very difficult and controversial because hemocyte types vary greatly depending on the insect species [3], [18]. It is important for scientists to devote more effort to the identification of hemocyte types in insects, especially immune-related hemocytes.

Several studies have shown that the total circulating hemocyte number (THC) is increased by immune challenge in insects, as we observed in Fig 2 [22], [23], [24]. Although our experiment did not directly address the source of the hemocytes (for example, a discrete hematopoietic organ or the replication of a progenitor cell type known as prohemocytes), this increasing hemocyte number may be related to the replication of a progenitor cell type, since we frequently observed the replication of prohemocytes in challenged larvae. Recently, we reported a relationship between hemocyte number and mitosis of prohemocytes in larvae of *Protaetia brevitarsis seulensis* [3]. With respect to the differential hemocyte number (DHC), the percentage of adiphohemocytes was specifically increased in immunized larvae. These results suggested that mitosis of prohemocytes or proliferation of adipohemocytes might be associated with immune reactions. Current studies are addressing the reason that the percentage of adipohemocytes substantially increased the during infection process.

After delivery of immune stimuli, the granulocytes were filled with relatively large granules. These granules may have resulted from fusion with lysosomes to carry out the degradation of pathogens. As shown in Fig 3A and 3B, only granulocyte granules were strongly stained by LysoTracker Red, which marks acidified compartments (usually lysosomes) in cells, and the red fluorescent signal was quantified by flow cytometry. The red fluorescent signal peaked at 4 h post-infection with bacteria. Assays of the phagocytic processing time are used to measure the function of the innate immune system in multicellular organisms. Klippel and Bilitewski (2007) showed that the phagocytic ability of macrophages is measured by incubating the phagocytes with fluorescently labeled foreign material for a defined period of time, ranging from several minutes to several hours [25]. In addition, we also showed that phagocytosis of insect hemocytes depended on the foreign particle size [3]. Likewise, the granulocytes in this insect also require time to engulf foreign particles. Furthermore, recent studies showed that autophagy has a dynamic relationship with phagocytosis [3], [26], [27], [28]. As described in detail previously [3], Fig 5 indicated that the uptake of microorganisms by granulocytes rapidly triggers the translocation of LC3 to the phagosome, and this mechanism helps to degrade intracellular hazardous substances and enhances pathogen killing. Our results reveal the existence of autophagic-phagocytic hybrid processes in this beetle larva [3]. Although autophagy was originally described as a starvation response of the cell, it may have innate and adaptive immune effector functions by facilitating pathogen detection and mediating pathogen clearance [3], [29], [30], [31], [32].

## Conclusions

In summary, six morphological types of circulating hemocytes were identified in larvae of the Japanese rhinoceros beetle *A*. *dichotoma*. The six hemocyte types were classified as prohemocytes, oenocytoids, adipohemocytes, spherulocytes, plasmatocytes, and granulocytes by microscopy. In addition, we demonstrated that an immunological challenge elicited morphological changes in granulocytes, including formation of phagolysosomes associated **wi**th autophagy.

## Materials and Methods

### Insect

The procedures including sample preparation, infection, and image analysis were performed as previously described [3]. The larvae of the Japanese rhinoceros beetle *Allomyrina dichotoma* (Linnaeus) (Coleoptera: Scarabaeidae) were reared in a constant environment incubator (MIR-553; Sanyo Electric Biomedical, Japan) at 60–70% relative humidity, 25±1°C temperature condition, and a 16h light: 8h dark cycle under aseptic conditions.

### Pathogenic microbial infection

To culture of bacteria and fungi, *Escherichia coli* and *Saccharomyces cerevisiae* were incubated overnight at 37°C in Luria-Bertani’s rich nutrient medium (LB broth) and at 25°C in yeast extract-peptone-dextrose (YPD) medium. The cold-anesthetized larvae were shallowly inserted into the dorsal vessel with *E*. *coli* and *S*. *cerevisiae* by a finely pulled glass needle (Haematokrit-kapillaren) (40 μl of 2 × 10^7^ for *E*. *coli* and 4 × 10^6^ for *S*. *cerevisiae*). To explore the competence of phagocytes *in vivo*, we used Green Florescent Protein (GFP)-expressing *E*.*coli* (modified DH5a) and yeast (GFP-tagged YDR385W, Invitrogen, San Diego, CA). All Cultures were normalized to OD_600_ = 4 for bacteria and OD_600_ = 4 for yeast using a spectrophotometer (Eppendorf AG, Hamburg, Germany) prior to being injected into the larvae. Hemocytes were collected and analyzed by puncture of the dorsal of each larva at 4, 8, 12, 24, 48h post infection to examine hemocyte morphology, including phagocytosis, nodulation, and quantity of the hemocytes. Experiments were replicated using independently collected blood of larvae.

### Counting of hemocytes

Hemolymph were extracted from dorsal blood vessel using a finely pulled glass needle (Haematokrit-kapillaren). Then, the hemolymph sample were placed into anti-coagulant solution (98mM NaOH, 186mM NaCl, 17mM EDTA, and 41mM citric acid (pH 4.5)) and mixed well. The mixed samples were centrifuged at 1,000 g for 10 minutes. After centrifugation, the plasma were removed and then hemocytes were washed with anti-coagulant solution used for *in vitro* experiments and hemocyte slide preparation. For the Total Hemocyte Counts (THC) and Differential Hemocyte Counts (DHC), hemocytes were placed in a sterile disposable hemocytometer slide (Ncubauer Improve, iNCYTO C-Chip DHC-N01. www.incyto.com) (10 μl capacity). The THC and DHC were counted using a light microscope (Leica DMI 3000B; 40× objective). Three independent experiments at 2, 4, 6, 8, 12 and 24h post infection with bacteria and fungi were performed for the THC and DHC. Thus, total 39 larvae were used to confirm the THC and DHC and the proportion of each hemocyte population was expressed as the Mean (roughly over 30,000 hemocytes, ± SEM).

### Hemocytes staining and visualization

Hemocytes staining was performed in control and challenged larvae which were infected with bacteria and fungi. For the hemocytes fixation, hemocytes were treated with 4% formaldehyde in PBS for 10 minutes at room temperature and were washed twice with anti-coagulant solution. To label of hemocyte cytoskeleton and nucleus, the fixed hemocytes were stained with fluorescently-conjugated phalloidin for F-actin cytoskeleton staining for 30 minutes and 4'-6-diamidino-2 Phenylindole for nucleus staining for 10 minutes (6 nM for F-actin and 5 μg/ml for DAPI). To test of lysosomes activity, hemocytes were stained with the acidotropic dye LysoTracker Red for 30 minutes at room temperature, washed three times with PBS, fixed with 4% paraformaldehyde for 15 minutes, washed again three times with PBS, and mounted (7.5mM; Molecular Probes). To evaluate the autophagosomes in granulocytes, hemocytes were labeled with Cyto-ID Autophagy Green dye reagent which enables detection and quantification of autophagic vacuoles (Cyto-ID Autophagy Detection Kit, Enzo). Relative percentage of autophagy were analyzed by flow cytometry and visualization of stained granulocyte were confirmed by fluorescence microscopes at 4, 8, 12 and 24h post infection, including experiments with control larva hemocytes.

### Microscopy, image processing, FACs analysis, and statistical analysis

All samples were observed with Leica DM2500 upright and DMI 3000B inverted fluorescence microscopes. Images of hemocyte morphology were acquired with Leica photo camera (2048 × 1536 pixels resolution) using the LMD application software version 4.1. The confocal microscope images were taken and analyzed with the aid of an Olympus FV1000 confocal microscope and Olympus image application software. For transmission electron microscope (TEM), the fixed hemocytes were post-fixed for 1 h at room temperature with 1% OsO_4_/0.8% potassium ferrycyanide, 5mM CaCl_2_ in 0.1M cacodylate buffer, PH 7.2. The sample were dehydrate in acetone and embedded in Epon. Ultrathinsection were generated by an ultramicrotome Pabisch Top Ultra 150 and collected on 200-mesh nickel grids. Thin section were stained with uranyl acetate and lead citrate and observed with a TEM Philips EM208. The FACS results were obtained and analyzed from 10,000 hemocytes per sample, by using FL1 (530/30 band-pass) channel for green fluorescence and FL3 (610/20 band-pass) channel for red fluorescence. All samples were sorted and analyzed by a BD FACSCanto flow cytometer and sample analysis was performed according to protocols developed for application using FACSDiva software from BD Biosciences (BD Bioscience; San Jose, CA). For statistical tests, we used values by Student’s two-tailed t-test or one-way ANOVA at a probability (P) value of less than 5%.
